# Biomarkers extracted by fully automated body composition analysis from chest CT correlate with SARS-CoV-2 outcome severity

**DOI:** 10.1038/s41598-022-20419-w

**Published:** 2022-09-30

**Authors:** René Hosch, Simone Kattner, Marc Moritz Berger, Thorsten Brenner, Johannes Haubold, Jens Kleesiek, Sven Koitka, Lennard Kroll, Anisa Kureishi, Nils Flaschel, Felix Nensa

**Affiliations:** 1 Institute of Diagnostic and Interventional Radiology and Neuroradiology, University Hospital Essen, Hufelandstraße 55, 45147 Essen, Germany; 2 Institute for Artificial Intelligence in Medicine (IKIM), University Hospital Essen, Girardetstraße 2, 45131 Essen, Germany; 3 Department of Anesthesiology and Intensive Care Medicine, University Hospital Essen, University Duisburg-Essen, Essen, Germany

**Keywords:** Viral infection, Prognostic markers

## Abstract

The complex process of manual biomarker extraction from body composition analysis (BCA) has far restricted the analysis of SARS-CoV-2 outcomes to small patient cohorts and a limited number of tissue types. We investigate the association of two BCA-based biomarkers with the development of severe SARS-CoV-2 infections for 918 patients (354 female, 564 male) regarding disease severity and mortality (186 deceased). Multiple tissues, such as muscle, bone, or adipose tissue are used and acquired with a deep-learning-based, fully-automated BCA from computed tomography images of the chest. The BCA features and markers were univariately analyzed with a Shapiro–Wilk and two-sided Mann–Whitney-U test. In a multivariate approach, obtained markers were adjusted by a defined set of laboratory parameters promoted by other studies. Subsequently, the relationship between the markers and two endpoints, namely severity and mortality, was investigated with regard to statistical significance. The univariate approach showed that the muscle volume was significant for female (*p*_severity_ ≤ 0.001, *p*_mortality_ ≤ 0.0001) and male patients (*p*_severity_ = 0.018, *p*_mortality_ ≤ 0.0001) regarding the severity and mortality endpoints. For male patients, the intra- and intermuscular adipose tissue (IMAT) (*p* ≤ 0.0001), epicardial adipose tissue (EAT) (*p* ≤ 0.001) and pericardial adipose tissue (PAT) (*p* ≤ 0.0001) were significant regarding the severity outcome. With the mortality outcome, muscle (*p* ≤ 0.0001), IMAT (*p* ≤ 0.001), EAT (*p* = 0.011) and PAT (*p* = 0.003) remained significant. For female patients, bone (*p* ≤ 0.001), IMAT (*p* = 0.032) and PAT (*p* = 0.047) were significant in univariate analyses regarding the severity and bone (*p* = 0.005) regarding the mortality. Furthermore, the defined sarcopenia marker (*p* ≤ 0.0001, for female and male) was significant for both endpoints. The cardiac marker was significant for severity (p_female_ = 0.014, p_male_ ≤ 0.0001) and for mortality (p_female_ ≤ 0.0001, p_male_ ≤ 0.0001) endpoint for both genders. The multivariate logistic regression showed that the sarcopenia marker was significant (*p*_severity_ = 0.006, *p*_mortality_ = 0.002) for both endpoints (OR_severity_ = 0.42, 95% CI_severity_: 0.23–0.78, OR_mortality_ = 0.34, 95% CI_mortality_: 0.17–0.67). The cardiac marker showed significance (p = 0.018) only for the severity endpoint (OR = 1.42, 95% CI 1.06–1.90). The association between BCA-based sarcopenia and cardiac biomarkers and disease severity and mortality suggests that these biomarkers can contribute to the risk stratification of SARS-CoV-2 patients. Patients with a higher cardiac marker and a lower sarcopenia marker are at risk for a severe course or death. Whether those biomarkers hold similar importance for other pneumonia-related diseases requires further investigation.

## Introduction

After its emergence in late 2019, SARS-CoV-2 quickly developed into a global pandemic with nearly 450 million infections and over 6 million deaths (03/2022)^1,2^. Whether an infection leads to a severe course of disease resulting in mechanical ventilation or death depends on numerous parameters and conditions. Several recent studies identified clinical parameters associated with a higher vulnerability to a severe clinical outcome^3–5^. These studies used general characteristics like age, gender, and Body Mass Index (BMI) combined with different clinical laboratory features to better understand the importance of such features when predicting the clinical course severity of individual SARS-CoV-2 patients^3,6–10^.

Recently published studies focused primarily on BMI as a predictive feature for clinical outcomes^3,6,7^. Sattar et al. showed that younger SARS-CoV-2 patients with an increased BMI were at higher risk of hospitalization and ICU transfer^3^. Within a systematic review, Huang et al. investigated studies that analyzed the impact of BMI on the clinical course and showed that multiple studies suggested BMI is a relevant predictive feature^11^. One reason behind the frequent use of BMI is its convenient accessibility. However, a mentionable drawback is that the BMI can be understood as a very shallow approximation of body composition, as its interpretability can be negatively affected by physical anomalies or unusual body proportions regarding muscle and fat volumes^12^.

Based on this knowledge, the focus of research has shifted to the usage of more detailed features describing the body composition of patients. Chandarana et al. extracted visceral, subcutaneous and total adipose tissue features based on L3 axial slices and investigated the impact of those features on the severity^13^. Phan et al. investigated the impact of cardiac adipose tissues on the severity and mortality on diabetic patients^14^. Yang et al. measured BCA features on L3 axial slices and showed that a high visceral to subcutaneous adipose tissue ratio, skeletal muscle attenuation and a high intramuscular fat are decisive features regarding the severity^15^. These findings align with research results from other medical areas, such as oncology, in which body composition features are dominant predictors for clinical outcomes such as mortality^16–20^.

The major disadvantage of body composition analysis (BCA) in clinical routine is the time-consuming and impracticable manual feature extraction process^21^. Therefore, many common BCA methods are semi-automated and/or use only reference regions for assessment, like the lumbar vertebra (L3)^13,15,20,22,23^. However, a 2D reference image at the level of L3 is also known to be only a rough estimation of the tissue composition, which may differ throughout the volume^24^. Furthermore, the L3 region is often not captured on a regular chest CT, rendering this method unsuitable for assessing patients with SARS-CoV-2 pneumonia in clinical routine.

We have leveraged a fully-automatic 3D semantic segmentation convolutional neural network (CNN) to overcome these limitations. This approach enables us to precisely quantify relevant body tissues like bone, muscle and multiple adipose tissues and combine them as potential predictive biomarkers to determine the clinical outcome of SARS-CoV-2 patients. This study aims to characterize the relationship between different tissue volumes automatically extracted from CT-thorax scans and the clinical outcome for admitted SARS-CoV-2 patients. Additionally, other relevant BCA biomarkers that can help predict the severity of a SARS-CoV-2 infection will be uncovered.

## Materials and methods

### Ethics declarations

This study was conducted in compliance with the guidelines of the Institutional Review Board of the University Hospital Essen (approval number 21-10029-BO). Due to the study's retrospective nature, the requirement of written informed consent was waived by the Institutional Review Board. The data were completely anonymized before being included in the study.

### Data

**Figure 1:**
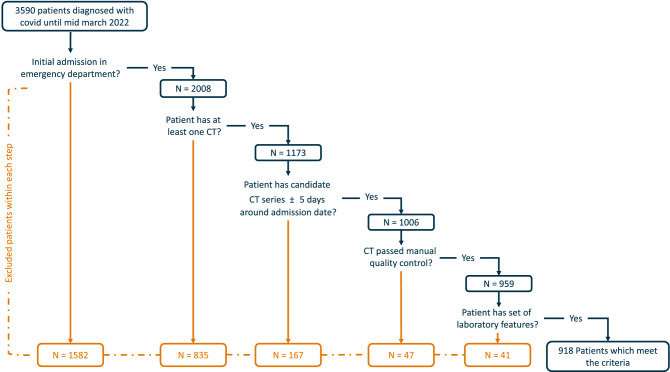
The data flow based on all identified and collected SARS-CoV-2 patients until March 2022. Overall, 3590 unique Patients were identified, but only 918 were used for further univariate and multivariate analysis as not all patients identified had a valid CT scan and/or the defined laboratory features available.

The retrospective data used for this study were collected at the University Hospital Essen. All patients with a positive SARS-CoV-2 diagnosis and an admission within the central emergency department between March 1st, 2020, and March 13th, 2022 were initially included. Subsequently, only patients with a valid CT thorax scan were considered. In the context of this study, a valid CT is defined as a CT-thorax scan taken as close to the timestamp of the admission as possible with a max difference of ± 5 days. The clinical outcome is divided into two endpoints “severity” and “mortality”. The first endpoint, “severity”, consists of the classes “mild” and “severe”. The class severe contains all patients who required mechanical ventilation and/or did not survive. The class “mild” encompasses all patients who survived without mechanical ventilation. The second endpoint, “mortality”, consists of the classes “survived” and “deceased” patients during their hospital stay. In addition to the imaging data, the following clinical parameters were included: age, gender, c-reactive protein (CRP)(mg/dl), leukocytes (/nl), hemoglobin (g/dl), and alanine aminotransferase (ALAT) (U/l). Furthermore, all CT scans were controlled manually, and scans with a low image quality or image artifacts were excluded. The final cohort contains all patients who meet the CT criteria and have all defined laboratory features available. The complete selection process for the cohort is visualized in Fig. 1. For further cohort characterization, a list of all comorbidities of included patients was extracted and enclosed within the supplemental material (Table S3).

As depicted in the visualized selection process, a dataset with 918 patients was assembled. CT scans taken both before and closest to the hospital admission date were selected for each patient. This resulted in a mean difference of 1.05 ± 0.82 days between the initial diagnosis date and the selected chest CT scan. The mean difference in days between the collected laboratory values and the patient admission was 0.71 ± 3.38 days.

### CT acquisition

The extracted 918 CT scans were examined with Siemens Somatom CT scanners which uses syngo CT VA50A (619), syngo CT VB20A (257), syngo VA48A (37) and syngo CT 2012B (5) software. All patients were scanned using the head-first-supine position and voxel spacings ranged from 0.54 to 0.97. In addition, 460 CT scans were performed using a pulmonary protocol. For the usage within the body composition network, all scans were resampled to a slice thickness of 5 mm.

### Body composition analysis

**Figure 2:**
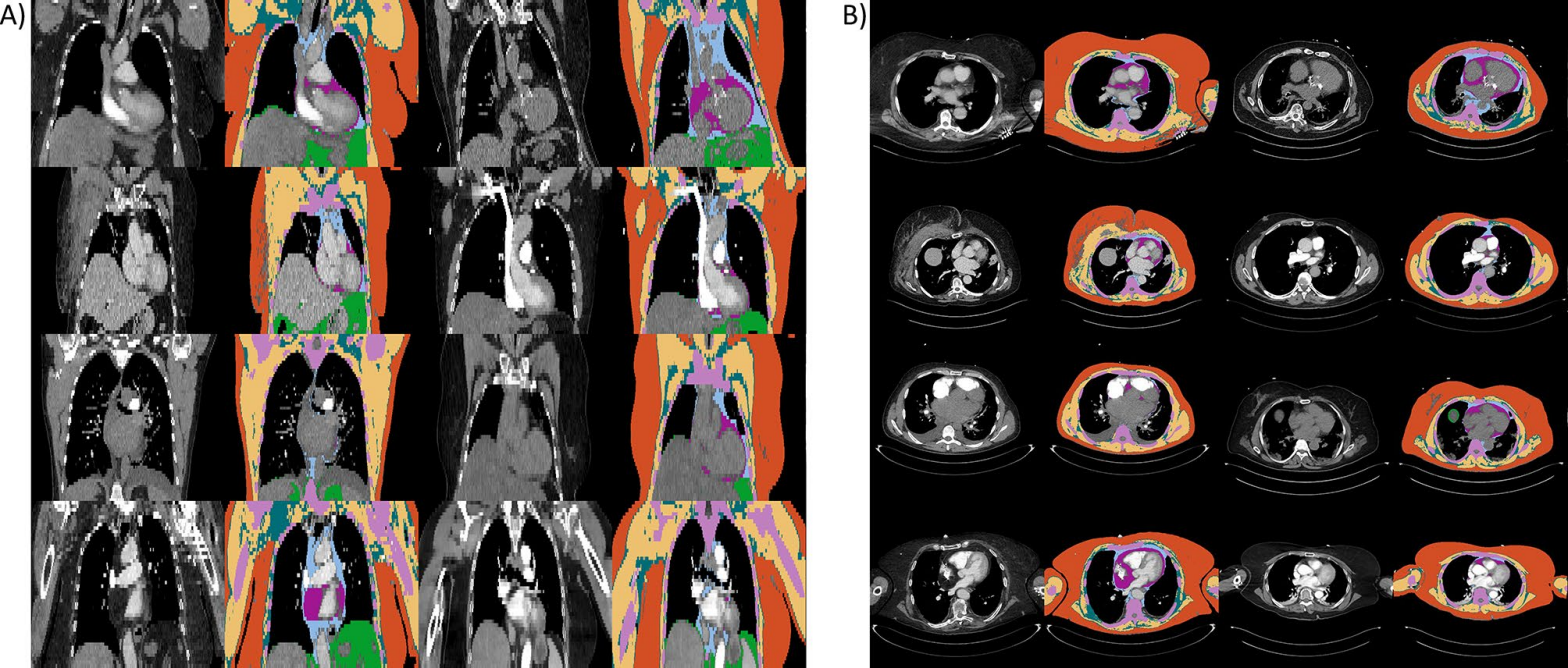
(**A**) Exemplary outputs of the BCA model in coronal view obtained from randomly selected patients. (**B**) Visualization of the BCA segmentations in the axial view for randomly selected patients. The tissue color codes are defined as follows**:** orange: subcutaneous adipose tissue, yellow: muscle tissue, cyan: intra- and intermuscular adipose tissue, pink: bone, light blue: pericardial adipose tissue, purple: epicardial adipose tissue, green: visceral adipose tissue.

The extraction of body composition features is based on a pre-trained convolutional neural network (CNN) published by Koitka et al.^21^. For this study, an enhanced approach of the proposed network to cover more body regions was used to generate the BCA segmentations which contain the following classes: bone, muscle, subcutaneous adipose tissue (SAT), visceral adipose tissue (VAT), intra- and intermuscular adipose tissue (IMAT), epicardial adipose tissue (EAT) and pericardial adipose tissue (PAT). Adipose tissues were derived using HU-based thresholding (− 190 to − 30 HU) from surrounding and detected body regions such as the dermis, abdomen, thorax, mediastinum, and pericardium. Muscle tissue was derived using a threshold of − 29 to 150 HU^21^. PAT and EAT, which are jointly referred to as pericardial fat, were subtracted from the latter two regions. Pericardial fat is located in the mediastinum adjacent to the parietal pericardium and surrounds the perivascular space of the coronary arteries. Epicardial fat is defined as a visceral fat deposit below the parietal pericardium. It directly surrounds and shares microcirculation with the myocardium. EAT and PAT, therefore, must be viewed separately and can also be distinguished embryologically^25–28^. Figure 2 visualizes a collage of coronal visualized segmentation results for randomly selected CT scans from the proposed cohort.

The model divides CT-thorax scans into four anatomical subgroups: whole scan, thoracic cavity, mediastinum, and pericardium. The tissue volumes were calculated using the thoracic cavity region from the provided CT-thorax scans. This enables overall comparability between all scans used for feature extraction. The BCA network defines the thoracic cavity as the chamber enclosed by the rib cage ranging from the superior thoracic aperture to the thoracic diaphragm and includes the trachea up to the cricoid cartilage. The raw BCA features were further normalized using the number of detected slices belonging to the thoracic cavity region to counteract differences in patient size or scan range. The normalized BCA features were further used to create two biomarkers: sarcopenia marker (SM) and Cardiac Marker (CM). The markers are defined as follows:

$$Sarcopenia \; Marker = \frac{Muscle}{Intra- and\;Intermuscular \; Adipose \; Tissue + Bone}$$

$$Cardiac \; Marker = \frac{Epicardial \; Adipose \; Tissue + Pericardial \; Adipose \; Tissue}{Muscle}$$

The SM uses the raw quantification of muscle divided by IMAT plus bone. As such, regarding patients mortality, it reflects the important ratio of muscle mass to skeletal volume^29^ and includes intramuscular adipose tissue as an additional risk factor^30^. A low score corresponds to a low muscle volume in relation to the skeletal volume and the volume of IMAT. The CM uses the adipose tissues of the heart (EAT and PAT) divided by the muscle volume. EAT and PAT are important risk factors for cardiac health^31–33^ and provide independent additional information to the calcium score^25^. By normalizing EAT and PAT to the muscle volume, on the one hand, the inter-individual comparability is increased, and on the other hand, the muscular habitus is included as a positive determinant for cardiovascular health^34^. For a visual interpretation of the markers, exemplary axial slices from patients with the highest and lowest marker values are visualized within the supplemental material (Fig. S3).

### Statistical analysis

Initially, all relevant BCA raw features for the marker creation (muscle, bone, IMAT, EAT, and PAT) were analysed univariately. In addition, for all features, the normality assumption was checked using the Shapiro–Wilk-Test^35^. For general reporting, the median and interquartile range (IQR) were calculated for all non-normally distributed continuous variables (median [IQR]). Mean and standard deviation (SD) were used to report normal distributed continuous variables (mean ± SD). Categorical variables were also reported as total count and percentages. For univariate testing of the BCA features, markers, and laboratory values a two-sided Mann–Whitney-U test^36^ was conducted.

Furthermore, a binary logistic regression was performed to estimate the effect of selected features on the severity and mortality. In the beginning, the skewness of the BCA and laboratory features were calculated. Since all features were highly skewed, all features were log2 transformed to counteract a skewed distribution. In addition, the correlation between the resulting sarcopenia and cardiac marker was calculated using Pearson's correlation index. Due to the high negative correlation of both markers, the logistic regression was conducted for each marker and adjusted to the following clinical features: CRP, leukocytes, hemoglobin, ALAT, age, and sex. It must be stated that the logistic regression was only used for statistical assessment of the defined BCA markers and not in a predictive way. The logistic regression results are described in adjusted odds ratios (OR), corresponding confidence intervals (95% CI), and resulting p-values. All calculated p-values within this study were rounded to three decimal points and values smaller than 0.001 or 0.0001 were reported as ≤ 0.001 and ≤ 0.0001.

The univariate and multivariate analysis was conducted based on the groups “mild” and “severe” for the severity and the classes “survived” and “deceased” for the mortality endpoint. The statistical analysis was performed using the python package statsmodels^37^ (logistic regression) and scipy^38^ (Shapiro–Wilk, Mann–Whitney-U).

### Approval for human experiments

The experiments of the study were approved by the Institutional Review Board of the University Hospital Essen (approval number 21-10029-BO). The experiments were performed in accordance with the declaration of Helsinki and with all guidelines set forth by the approving institutional review board. Due to the study's retrospective nature, the requirement of written informed consent was waived by the Institutional Review Board. The data were completely anonymized before being included in the study.

## Results

The division within the gender groups resulted in a subcohort of 354 female patients encompassing 239 mild and 115 severe cases (77 deceased) and 564 male patients encompassing 360 mild and 204 severe cases (109 deceased). Table 1 shows the baseline statistics of the demographic parameters and the laboratory features concerning the severity classes and mortality.

**Table 1:** Baseline patient characteristics, including the gender distribution and age statistics with respect to the severity and mortality endpoint within the cohort of 918 patients.

	Mild	Severe	Deceased
No. included	n = 599 (65%)	n = 319 (35%)	n = 186 (20%)
Gender	Female = 239 (40%) Male = 360 (60%)	Female = 115 (36%) Male = 204 (64%)	Female = 77 (41%) Male = 109 (59%)
Age (in years)	63 [25]	71 [23]	78 [18.75]
CRP (mg/dl)	5.30 [8.05]	12.20 [11.70]	11.70 [12.15]
Leukocytes (/nl)	6.07 [3.53]	7.99 [5.21]	8.15 [5.59]
Hemoglobin (g/dl)	13.20 [2.35]	12.70 [2.85]	12.15 [2.77]
ALAT (U/l)	29 [27]	33 [35]	30 [29.75]

The values for continuous variables are reported as median [IQR] and for categorical variables as total count and percentages.

The first analysis univariately compares the normalized BCA features bone, muscle, IMAT, EAT, and PAT, which will be used to obtain markers for each gender using a Mann–Whitney-U test. The obtained results are depicted in Table 2.

**Table 2:** Overview of the median volume [IQR] and p-values of the normalized BCA-Features (per slice) within both endpoint categories severity and mortality.

Tissue	Sex	Severity			Mortality		
		Average volume per slice [mL], mild cases	Average volume per slice [mL], severe cases	p-value	Average volume per slice [mL], survived cases	Average volume per slice [mL], deceased cases	p-value
Bone	Female	28 [4.04]	29 [3.93]	≤ 0.001	28 [4.28]	29 [4.68]	0.005
	Male	36 ± 4.21	37 ± 4.38	0.013	36 ± 4.17	37 ± 4.70	0.014
	Overall	33 [8.23]	34 [8.62]	≤ 0.001	33 [8.07]	34 [9.32]	0.104
Muscle	Female	49 [13.12]	44 [15.81]	≤ 0.001	49 [13.71]	40 [13.51]	≤ 0.0001
	Male	73 [26.37]	69 [25.59]	0.018	74 [27.16]	62 [21.74]	≤ 0.0001
	Overall	62 [29.24]	60 [29.85]	0.057	63 [30.30]	53 [25.27]	≤ 0.0001
IMAT	Female	20 [9.59]	21 [11.09]	0.032	20 [9.57]	21 [11.24]	0.079
	Male	18 [10.84]	22 [11.32]	≤ 0.0001	19 [10.87]	22 [10.61]	≤ 0.001
	Overall	19 [10.39]	22 [11.40]	≤ 0.0001	19 [10.53]	22 [10.80]	≤ 0.001
EAT	Female	1 [1.29]	1 [1.50]	0.709	1 [1.22]	2 [1.65]	0.201
	Male	2 [1.57]	2 [1.66]	≤ 0.001	2 [1.48]	2 [1.70]	0.011
	Overall	2 [1.46]	2 [1.55]	≤ 0.001	2 [1.44]	2 [1.65]	0.008
PAT	Female	3 [3.0]	4 [2.95]	0.047	4 [2.87]	4 [3.45]	0.075
	Male	6 [4.60]	7 [4.21]	≤ 0.0001	6 [4.45]	7 [4.53]	0.003
	Overall	5 [4.39]	6 [4.77]	≤ 0.0001	5 [4.37]	6 [4.97]	0.006

Only the male average bone volume per slice was normally distributed and thus is reported as mean and SD. The p-values were calculated using the Mann–Whitney-U test IMAT: intra- and intermuscular adipose tissue, EAT: epicardial adipose tissue, PAT: pericardial adipose tissue.

**Figure 3:**
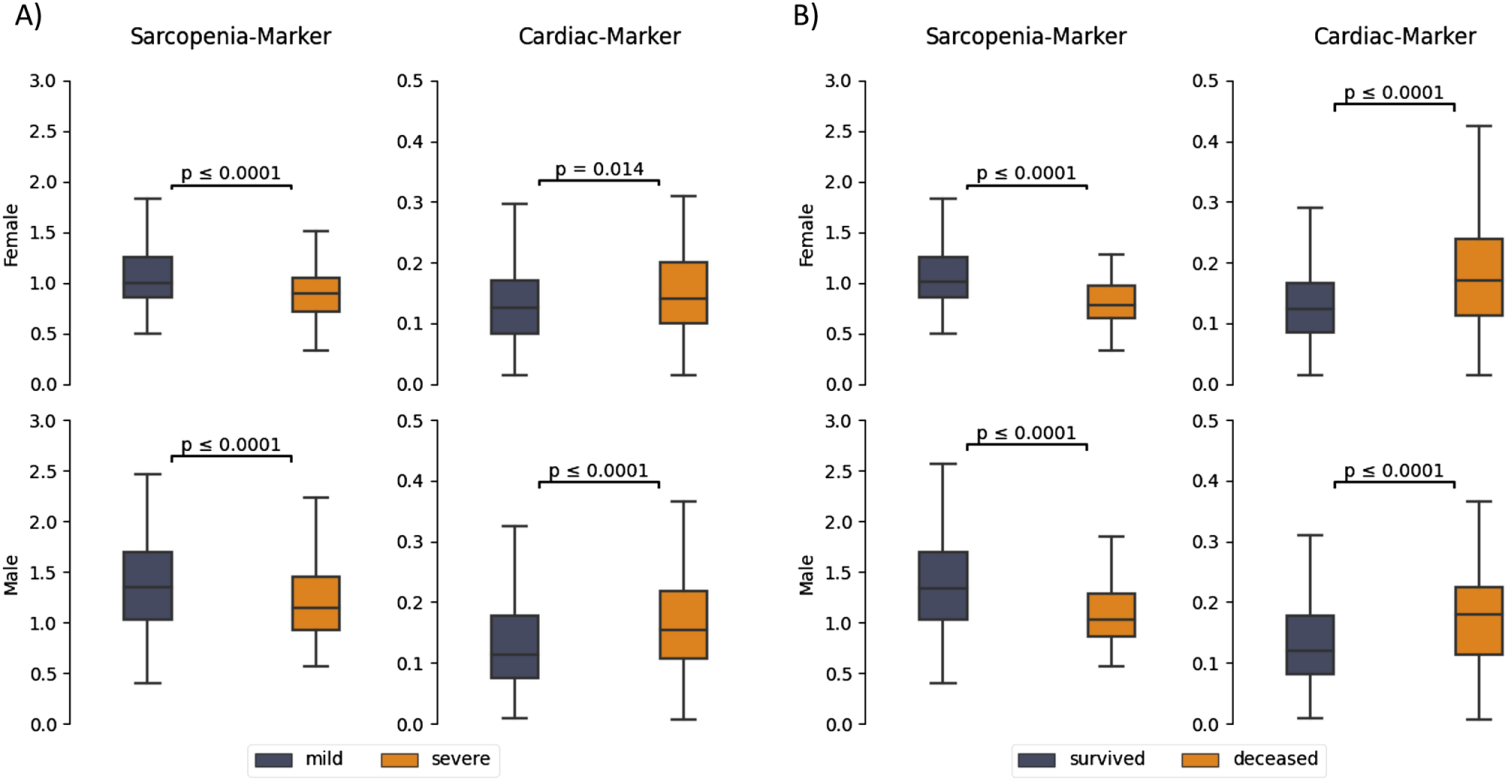
A box plot visualization including Mann–Whitney-U tests of the sarcopenia and cardiac marker for the female patients (first row) and male patients in the cohort (second row). (**A**) Univariate analysis regarding severity (**B**) Univariate analysis regarding mortality.

As the results of the univariate raw BCA feature analysis indicate, the selected features are mainly significant in the male patient group (muscle, bone, IMAT, EAT, and PAT). In contrast to that, muscle, bone, and IMAT were also significant for the female group. In addition to the results presented in Table 1, the corresponding boxplot visualizations are enclosed within the supplemental material (Figs. S1, S2). Furthermore, the univariate setting was also conducted for the laboratory features and is enclosed within the supplemental material (Table S1). Subsequently, BCA features were combined to generate the sarcopenia and cardiac marker which were tested univariately as well. The resulting significance of the tests are depicted in Fig. 3.

Based on the results of the Mann–Whitney-U Test, the SM (*p*_female_ ≤ 0.0001, *p*_male_ ≤ 0.0001) as well as the CM (*p*_female=_0.014, *p*_male_ ≤ 0.0001) show significance within the univariate setting regarding the severity endpoint. Furthermore, the SM (*p*_female_ ≤ 0.0001, *p*_male_ ≤ 0.0001) and CM (*p*_female_ ≤ 0.0001, *p*_male_ ≤ 0.0001) also showed significance regarding the mortality endpoint. In comparison to the BCA markers, the BMI was only available for 349 patients (122 female, 227 male) from which 64 were severe and 21 were deceased. Within the proposed univariate Setting it could be shown that the BMI was not significant within both groups. The complete results for the univariate analysis of the BMI are depicted in the supplemental material (Table S2).

Since the SM and CM are negatively correlated (r_severity_ = − 0.76, r_mortality_ = − 0.79) and share the muscle volume per definition, both markers were adjusted to the following clinical parameters for each gender group: CRP, leukocytes, hemoglobin, ALAT, and age. The resulting adjusted ORs and the corresponding p-values of the logistic regression are presented in two sections within Table 3, with section A referring to SM and section B referring to CM.

**Table 3:** Adjusted odds ratios including the 95% CI and the p-value for the severity and mortality endpoint for (**A**) sarcopenia marker and (**B**) cardiac marker.

(A)	Severity			Mortality		
Features	OR	95% CI	p-value	OR	95% CI	p-value
Sarcopenia marker	0.42	[0.23, 0.78]	0.006	0.34	[0.17, 0.67]	0.002
CRP	2.15	[1.79, 2.59]	≤ 0.0001	1.67	[1.36, 2.06]	≤ 0.0001
Leukocytes	1.60	[1.20, 2.15]	≤ 0.001	1.51	[1.08, 2.09]	0.015
Hemoglobin	0.19	[0.11, 0.30]	≤ 0.0001	0.10	[0.05, 0.17]	≤ 0.0001
ALAT	1.32	[1.05, 1.65]	0.016	1.14	[0.87, 1.47]	0.337
Age	1.01	[1.00, 1.01]	0.226	1.03	[1.02, 1.04]	≤ 0.0001
Sex	0.69	[0.49, 0.96]	0.027	0.74	[0.50, 1.08]	0.121

(B)	Severity			Mortality		
Features	OR	95% CI	p-value	OR	95% CI	p-value
Cardiac marker	1.42	[1.06, 1.89]	0.018	1.19	[0.85, 1.67]	0.311
CRP	2.16	[1.80, 2.59]	≤ 0.0001	1.69	[1.37, 2.07]	≤ 0.0001
Leukocytes	1.65	[1.22, 2.21]	≤ 0.001	1.51	[1.08, 2.09]	0.015
Hemoglobin	0.20	[0.11, 0.34]	≤ 0.0001	0.09	[0.04, 0.16]	≤ 0.0001
ALAT	1.31	[1.04, 1.64]	0.019	1.08	[0.83, 1.40]	0.538
Age	1.01	[1.00, 1.02]	0.018	1.05	[1.03, 1.05]	≤ 0.0001
Sex	0.85	[0.62, 1.17]	0.324	0.90	[0.62, 1.30]	0.577

**Figure 4:**
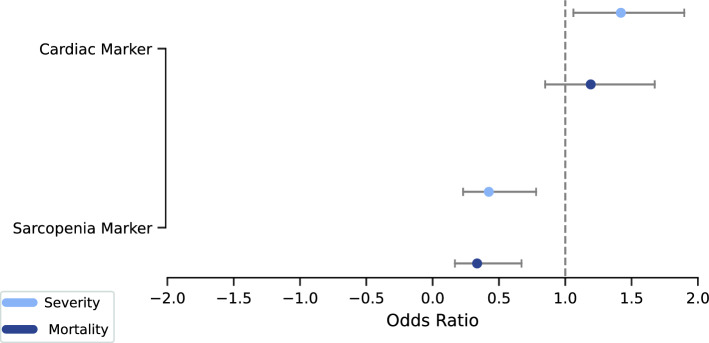
Odds ratio plot for the multivariate logistic regression conducted for both endpoints. The horizontal line is set to an odds ratio of 1, and the odds ratios are displayed with circles. Additionally, the confidence intervals (CI 5%, CI 95%) are presented with the left bars (5%) and right bars (95%).

Given the results of logistic regression, the sarcopenia (OR = 0.42, p = 0.006, 95% CI 0.23–0.78) and the cardiac marker (OR = 1.42, p = 0.018, 95% CI 1.06–1.90) are both significant regarding the severity endpoint. However, only the sarcopenia marker (OR = 0.34, p = 0.002, 95% CI: 0.17–0.67) is significant for the mortality endpoint. The ORs and the corresponding confidence intervals are presented in Fig. 4.

## Discussion

We investigated the significance of two defined markers, which were automatically extracted from tissues depicted in CT thoracic scans, for the clinical outcome of SARS CoV-2-patients. The main aim was to derive relevant BCA-based biomarkers that could be used as potential features for the risk stratification of SARS CoV-2 patients regarding two endpoints disease severity and mortality.

Our results indicate that BCA biomarkers are statistically related to the clinical course for SARS-CoV-2 patients. As indicated by the p-values from the univariate statistical test, the raw BCA features muscle and bone were substantial for both gender groups and endpoints. In addition, IMAT, PAT, and EAT were considerable for male patients for both endpoints. Furthermore, for female patients, IMAT and PAT were substantial regarding the severity endpoint. The combination of those raw BCA features into the defined sarcopenia and cardiac marker showed that those markers were univariately significant for both genders and both endpoints. Furthermore, the multivariate logistic regression showed that the sarcopenia marker is statistically significant in addition to proven clinical laboratory features for both endpoints. The cardiac marker was only significant regarding the severity endpoint. Based on the ORs we delineate the interpretation of both markers as follows: Patients exhibiting a high sarcopenia marker have lower odds of developing a severe course of disease or death. In contrast, patients with a high cardiac marker are associated with higher odds of developing a severe course or death. In the development of a new risk score for outcome prediction in patients with SARS-CoV-2, these biomarkers should be taken into account since a CT of the thorax is a standard procedure for SARS-CoV-2 patients and the extraction of tissue volumes can be performed conveniently and fully automated within the clinical routine, as shown by Koitka et al.^21^. In addition, the presented method and associated BCA features provide a much more accurate and diverse analysis of the patient's body than, for example, BMI. This was also shown by the univariate analysis performed (Supplemental Material Table S2).

These results tie well with other studies wherein the relation of different adipose tissues and/or the muscle volume with the clinical course of SARS-CoV-2 patients was shown. Chandarana et al. demonstrated that the VAT volume measured on an L3 region axial slice is a valuable feature for identifying SARS-CoV-2 patients in need of hospitalization^13^. Grodecki et al. outlined that EAT volume and attenuation seem to be associated with the quantitative burden of SARS-CoV-2 pneumonia and a larger EAT volume or attenuation might independently predict clinical deterioration or death^39^. Schiaffino et al. showed that patients with lower paravertebral muscle areas and attenuation have a higher risk of ICU admission^40^. Chandarana et al. used BCA features aggregated from an L3 axial slice to calculate muscle adipose tissue and muscle mass ratio and VAT to total adipose tissue ratios and showed that those features have a predictive value for the identification of patients with a need for hospitalization^22^.

Our results generally support these findings, which indicate that the adipose tissues located in the cardiac region and muscle volume are decisive features that correlate with the clinical outcome. By contrast, the sarcopenia marker performs well for both endpoints but best in context of the patient mortality. Unlike Grodecki et al. and Schiaffino et al., we deliberately did not include tissue density in our analysis because it depends on many technical parameters, such as tube voltage, hardening artifacts, reconstructed slice thickness and also contrast administration. A very high degree of standardization in image acquisition is therefore required, which is often not available in routine clinical practice and emergency examinations. In view of a practically applicable and robust method, we, therefore, restricted our study to tissue volumes as a foundation for the defined markers.

In contrast to the above-mentioned studies, our work presents a novel approach to quantify all body tissues within seconds, thus making BCA features readily available for use in clinical routine. To the best of our knowledge, none of the presented studies could quantify all BCA features for the complete thoracic cavity. Instead, most studies used reference axial L3 slices for the feature aggregation which is not applicable within the clinical routine since this region is not present in a thoracic CT. Our study presents a novel approach to quantifying all relevant tissues and using them for BCA-based biomarker aggregation. The results of our study suggest that sarcopenia and cardiac marker can also be added to the list of significant parameters. To access these non-invasive features, only a suitable CT scan that the BCA algorithm can automatically process is needed. The presented approach is equally suitable for both standard and emergency cases. From the medical point of view, the clinical application of the approach presented here depends on the existence of a CT thorax scan. In contrast, from a technical point of view, the application requires integrating the BCA network^21^. Consequently, only cases in which a CT scan is not performed cannot be assesed with this approach. These will most likely be mild cases rather than severe ones.

A notable limitation of this study is that within our dataset, information on SARS-CoV-2 mutations was unavailable at the time. Because the data underlying this study was collected from multiple SARS-CoV-2 waves (2020–2022), the effect of SARS-Cov-2 mutations could have influenced the likelihood of developing a severe disease course or mortality. Future studies should investigate the significance and importance of BCA features in the context of SARS-CoV-2 variants. A further limitation is the monocentric design of our study. Future studies would benefit from a multicentric approach, in which patients with diverse geographical regions of origin are included. This would strengthen the results and indicate the overall applicability of BCA feature extraction. It would also minimize potential biases and counteract the statistical influence of different SARS-CoV-2 mutations on the analysis.

In future studies, large patient cohorts should be used to investigate the applicability of BCA feature extraction in the determination of newly-admitted SARS-CoV-2 patients’ clinical courses. The applicability of BCA feature extraction to the clinical course predictions of other forms of pneumonia, influenza, and ARDS also warrants further investigation. For example, in the case of pneumonia, performing a fully automated BCA on hospitalized patients shortly after admission could potentially enable the early detection of severe disease courses. Implementing BCA in the clinical workflow could offer additional prognostic value by utilizing already gathered data (e.g., CT scans) without any additional expenditure of time or resources.

## Conclusion

The results of the study show that BCA markers based on raw BCA parameters extracted automatically from CT scans have the potential to improve risk stratification in patients with acute SARS-Cov-2 infection. Due to the robust and fully automatable methodology, these parameters should be considered in developing new risk scores. Future studies should investigate the predictive value of these features in relation to different SARS-CoV-2 mutation strands, as well as the applicability of BCA feature extraction in other acute respiratory conditions.

## Supplementary Information

Supplementary Information.

## Data Availability

The dataset is not publicly available. Reasonable requests should be directed to the corresponding author for consideration and can be provided pending appropriate institutional review board approvals.

## Abbreviations

ALAT: Alanine aminotransferase
BCA: Body composition analysis
CNN: Convolutional neural network
CRP: C-reactive protein
CI: Confidence interval
EAT: Epicardial adipose tissue
IMAT: Intra- and intermuscular adipose tissue
IQR: Interquartile range
PAT: Pericardial adipose tissue
SAT: Subcutaneous adipose tissue
SD: Standard deviation
VAT: Visceral adipose tissue
OR: Odds ratio
