# Supplementary material for: Biomarkers extracted by fully automated body composition analysis from chest CT correlate with SARS-CoV-2 outcome severity
Source: Sci Rep. 2022 Sep 30;12:16411. doi: 10.1038/s41598-022-20419-w (PMC9524347; doi:10.1038/s41598-022-20419-w)
Supplement: Supplementary file 1 — Supplementary Information. [file 41598_2022_20419_MOESM1_ESM.docx]

Biomarkers extracted by fully automated body composition analysis from chest CT correlate with SARS-CoV-2 outcome severity

René Hosch^1,2^, Simone Kattner^3^, Marc Moritz Berger^3^, Thorsten Brenner^3^, Johannes Haubold^1^, Jens Kleesiek^2^, Sven Koitka^1,2^, Lennard Kroll^1,2^, Anisa Kureishi^2^, Nils Flaschel^1,2^, Felix Nensa^1,2^

^1^ Institute of Diagnostic and Interventional Radiology and Neuroradiology, University Hospital Essen, Hufelandstraße 55, 45147, Essen, Germany

^2^ Institute for Artificial Intelligence in Medicine (IKIM), University Hospital Essen, Girardetstraße 2, 45131, Essen, Germany

^3^ Department of Anesthesiology and Intensive Care Medicine, University Hospital Essen, University Duisburg-Essen, Essen, Germany

# Supplemental Material:


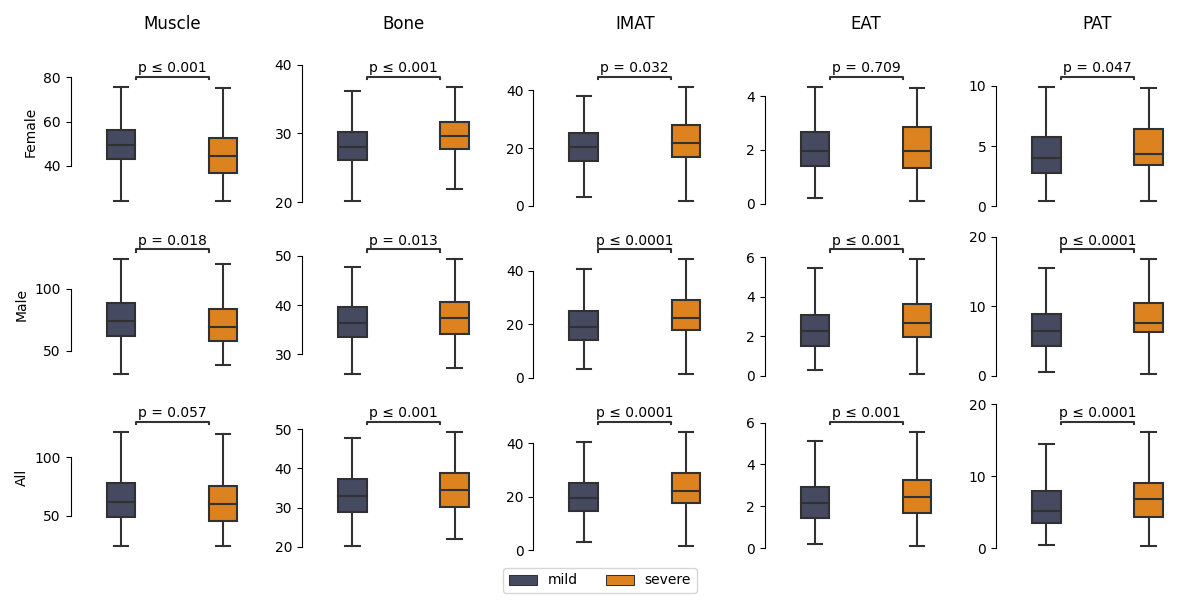


Figure S1: A box plot visualization and Mann-Whitney-U test of the normalized (average per slice) BCA feature volume for the female, male, and all patients regarding severity. IMAT: intra- and intermuscular adipose tissue, EAT: epicardial adipose tissue, PAT: pericardial adipose tissue.


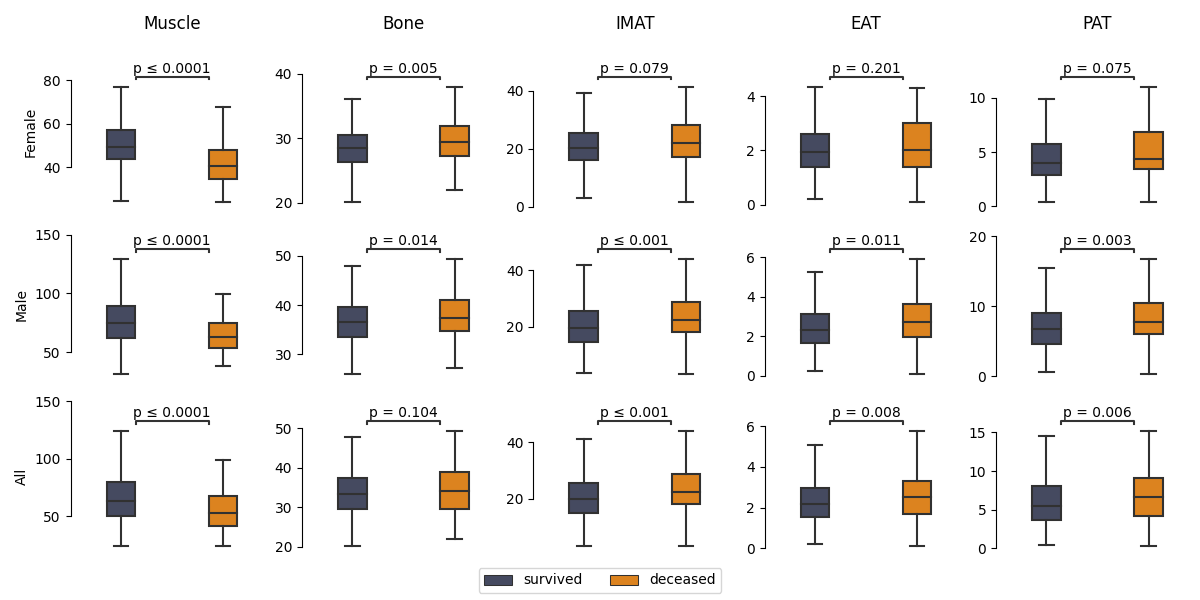


Figure S2: A box plot visualization and Mann-Whitney-U test of the normalized (average per slice) BCA feature volume for female, male, and all patients regarding mortality. IMAT: intra- and intermuscular adipose tissue, EAT: epicardial adipose tissue, PAT: pericardial adipose tissue.

##

**
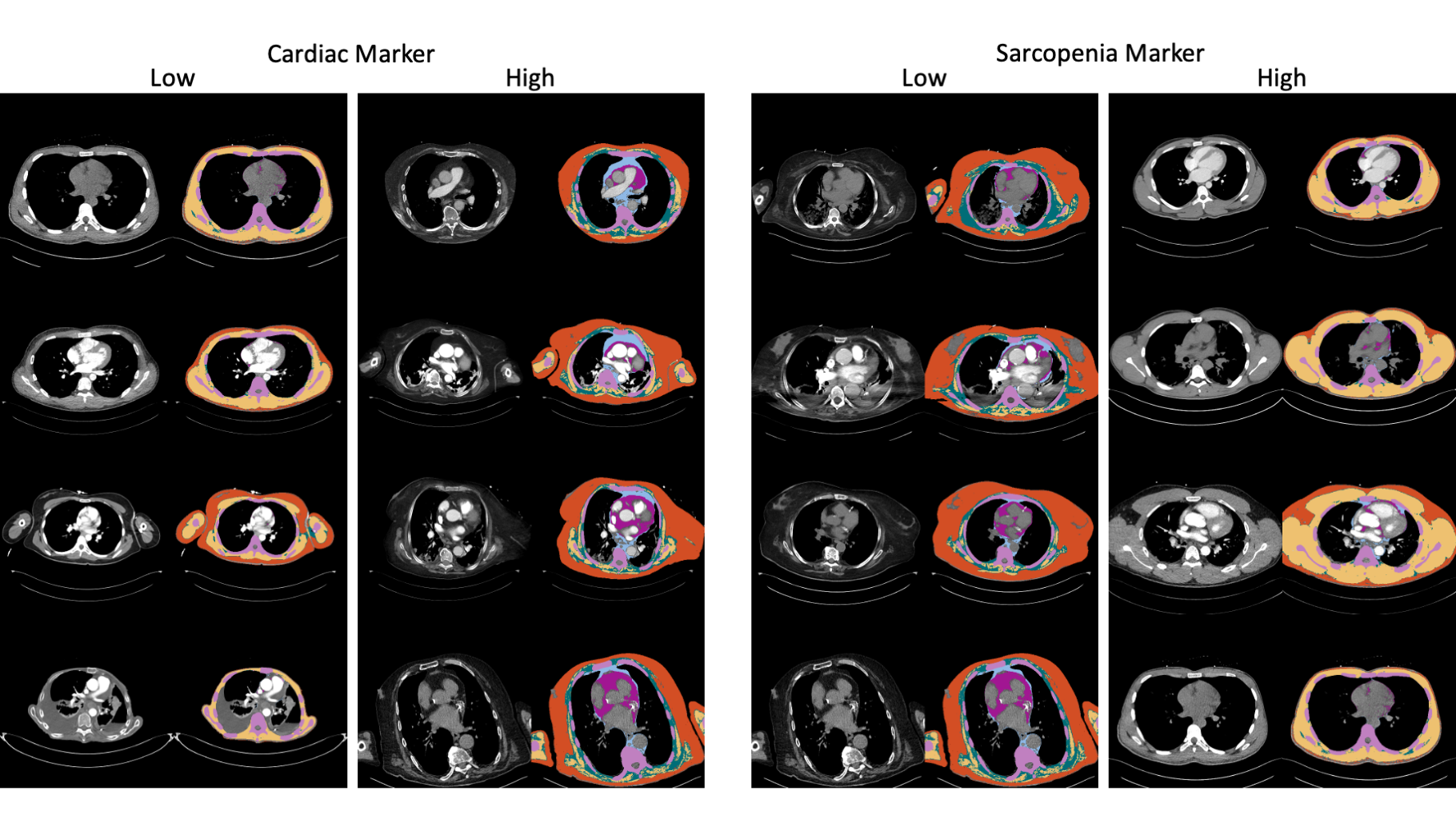
**

Figure S3: Exemplary outputs of the BCA model in axial view. For each marker, the four patients with the highest and lowest values were selected. The tissue color codes are defined as follows**:** orange: subcutaneous adipose tissue, yellow: muscle tissue, cyan: intra- and intermuscular adipose tissue, pink: bone, light blue: pericardial adipose tissue, purple: epicardial adipose tissue, green: visceral adipose tissue.

| Laboratory Value | Sex | Severity | | | Mortality | | |
| --- | --- | --- | --- | --- | --- | --- | --- |
|  |  | Median [IQR]  mild cases | Median [IQR], severe cases | p-value | Median  [IQR], survived cases | Median  [IQR], deceased cases | p-value |
| CRP (mg/dl) | Female | 4.70 [6.90] | 12.10 [10.55] | ≤0.0001 | 5.20 [8.10] | 11.90 [12.2] | ≤0.0001 |
|  | Male | 6.20 [8.52] | 12.30 [11.75] | ≤0.0001 | 7.10 [9.55] | 11.70 [12.3] | ≤0.0001 |
|  | Overall | 5.30 [8.05] | 12.20 [11.70] | ≤0.0001 | 6.35 [9.30] | 11.70 [ 12.15] | ≤0.0001 |
| LEUA (/nl) | Female | 6.32 [3.65] | 8.15 [5.70] | ≤0.0001 | 6.43 [3.77] | 8.56 [5.62] | ≤0.0001 |
|  | Male | 5.93 [3.50] | 7.80 [5.02] | ≤0.0001 | 6.27 [4.02] | 7.77 [5.16] | ≤0.0001 |
|  | Overall | 6.07 [3.53] | 7.99 [5.22] | ≤0.0001 | 6.32 [4.0] | 8.15 [5.59] | ≤0.0001 |
| HB (g/dl) | Female | 12.40 [2.40] | 11.80 [2.95] | 0.006 | 12.40 [2.50] | 11.70 [3.30] | 0.034 |
|  | Male | 13.80 [2.30] | 13.20 [2.50] | 0.002 | 13.80 [2.30] | 12.70 [2.60] | ≤0.0001 |
|  | Overall | 13.20 [2.35] | 12.70 [5.22] | ≤.001 | 13.20 [2.30] | 12.15 [2.77] | ≤0.0001 |
| ALAT (U/l) | Female | 26.0 [19.0] | 23.0 [22.0] | 0.443 | 26.0 [21.0] | 19.0 [18.0] | 0.015 |
|  | Male | 35.0 [30.25] | 37.0 [37.0] | 0.036 | 36.0 [32.0] | 36.0 [35.0] | 0.786 |
|  | Overall | 29.0 [27.0] | 33.0 [35.0] | 0.107 | 32.0 [29.0] | 30.0 [29.75] | 0.065 |

Table S1: Overview of the mean volume, standard deviation, and p-values of the extracted BCA-Features within both endpoint categories severity and mortality. The p-value was calculated using the Mann-Whitney-U test.

|  | Sex | Severity | | | Mortality | | |
| --- | --- | --- | --- | --- | --- | --- | --- |
|  |  | Median [IQR]  mild cases | Median [IQR], severe cases | p-value | Median  [IQR], survived cases | Median  [IQR], deceased cases | p-value |
| BMI (kg/m^2^) | Female | 29.3 [7.69] | 30.08 [8.24] | 0.39 | 29.82 [8.15] | 28.17 [5.83] | 0.12 |
|  | Male | 27.77 [5.40] | 27.76 [5.04] | 0.37 | 27.77 [5.37] | 25.9 [6.17] | 0.13 |
|  | Overall | 28.08 [6.40] | 28.21 [5.86] | 0.43 | 28.19 [6.34] | 27.68 [6.17] | 0.07 |

Table S2: Univariate comparison of the patients with an available BMI +/-5 days around the corresponding CT scan. The conducted Mann-Whitney-U tests show, that there is no significance between the groups regarding both endpoints. The BMI is reported as the median and IQR for all groups.

| Disease | Total (n=918) | Mild (n=599) | Severe (n=319) | Deceased (n=186) |
| --- | --- | --- | --- | --- |
| Hypertension | 258 (28%) | 152 (25%) | 106 (33%) | 76 (41%) |
| Diabetes | 113 (12%) | 66 (11%) | 47 (15%) | 37 (20%) |
| COPD | 52 (6%) | 30 (5%) | 22 (7%) | 12 (6%) |
| Asthma | 14 (2%) | 11 (2%) | 3 (< 1%) | 3 (2%) |
| Cardiovascular Disease | 274 (30%) | 155 (26%) | 119 (37%) | 86 (46%) |
| Liver Disease | 37 (4%) | 22 (4%) | 15 (5%) | 10 (5%) |
| Neoplasm | 223 (24%) | 133 (22%) | 90 (28%) | 63 (39%) |
| Renal Disorder | 116 (13%) | 64 (16%) | 52 (16%) | 41 (22%) |

Table S3: List of present comorbidities within the extracted patient collective grouped by the severity categories mild and severe and the deceased category. All values are presented as total counts and percentages for each group.
